# Case Report: Ectopic Adrenocortical Carcinoma in the Ovary

**DOI:** 10.3389/fendo.2021.662377

**Published:** 2021-03-19

**Authors:** Wen-Hsuan Tsai, Tze-Chien Chen, Shuen-Han Dai, Yi-Hong Zeng

**Affiliations:** ^1^ Division of Endocrinology and Metabolism, Department of Internal Medicine, Mackay Memorial Hospital, Taipei, Taiwan; ^2^ Department of Obstetrics and Gynecology, Mackay Memorial Hospital, Taipei, Taiwan; ^3^ Department of Pathology, Mackay Memorial Hospital, Taipei, Taiwan; ^4^ Department of Medicine, MacKay Medical College, New Taipei City, Taiwan

**Keywords:** ectopic, adrenocortical carcinoma, ovary, immunohistochemistry, metastasis

## Abstract

Adrenocortical carcinoma (ACC) is a rare malignancy with an incidence of 0.7–2.0 cases/million habitants/year. ACCs are rare and usually endocrinologically functional. We present the case of a 59-year-old woman who experienced abdominal fullness for 6 months and increased abdominal circumference. A large pelvic tumor was observed. She underwent cytoreductive surgery and the pathological test results revealed local tumor necrosis and prominent lympho-vascular invasion. Neuroendocrine carcinoma was the first impression, but positivity for synaptophysin, alpha-inhibin, transcription factor enhancer 3 (TFE-3), calretinin (focal), and CD56 (focal) and high Ki-67-labeling proliferating index (>80%) confirmed the diagnosis of ectopic ACC. Ectopic primary aldosteronism could not be excluded. However, we did not perform saline infusion test or captopril test due to poor performance status. When pathological test reports reveal neuroendocrine features not typically found in the organ being examined, IHC staining should be performed to rule out ectopic ACC. Whether the ectopic ACC is functional or not requires complete survey.

## Introduction

Adrenocortical carcinoma (ACC) is a rare malignancy with an incidence of 0.7–2.0 cases/million habitants/year. It occurs at any age, with two peaks in the first decade of life and between 40 and 50 years. Women are frequently affected (55–60%) (1). The adrenal glands are of dual embryological origin. The adrenal cortex is derived from the coelomic mesoderm of the urogenital ridge, and the adrenal medulla arises from the neural crest tissue (2). Ectopic adrenal rests exist along the migration path of adrenal cortex development, and these anatomic sites include the celiac plexus, kidney, ovary, broad ligament, testis, and spermatic cord (3, 4). The brain, lungs, and stomach have been reported to be rare sites of ectopic rests (3, 4). Ectopic adrenal tissue is found in 50% neonates, and most of the ectopic rests undergo atrophy (5). The occurrence of adrenal rest tissue in adults is 1% (6).

Tumors arising from adrenal rests are uncommon and most of them are functional, resulting in endocrinopathy diagnosed pre-operatively (7). Non-functional tumors are also uncommon and are usually discovered incidentally or during autopsy (7). Malignancies arising from adrenal rests are extremely rare with very few cases reported (8), showing a mean patient age of 36.4 years (0.4–65 years) and equal sex distribution (female:male ratio, 7:6). Studies have reported tumors located in the retroperitoneum (n = 5), testis/scrotum (n = 3), liver (n = 2), kidney (n = 1), spinal cord (n = 1), and pelvis (n = 1); 62% of the tumors were functional, with Cushing’s syndrome as the most common presentation (8). Herein, we present the case of a 59-year-old woman with ectopic ACC in the ovary. Consent has not been obtained because the patient is deceased.

## Case Report

**Figure 1:**
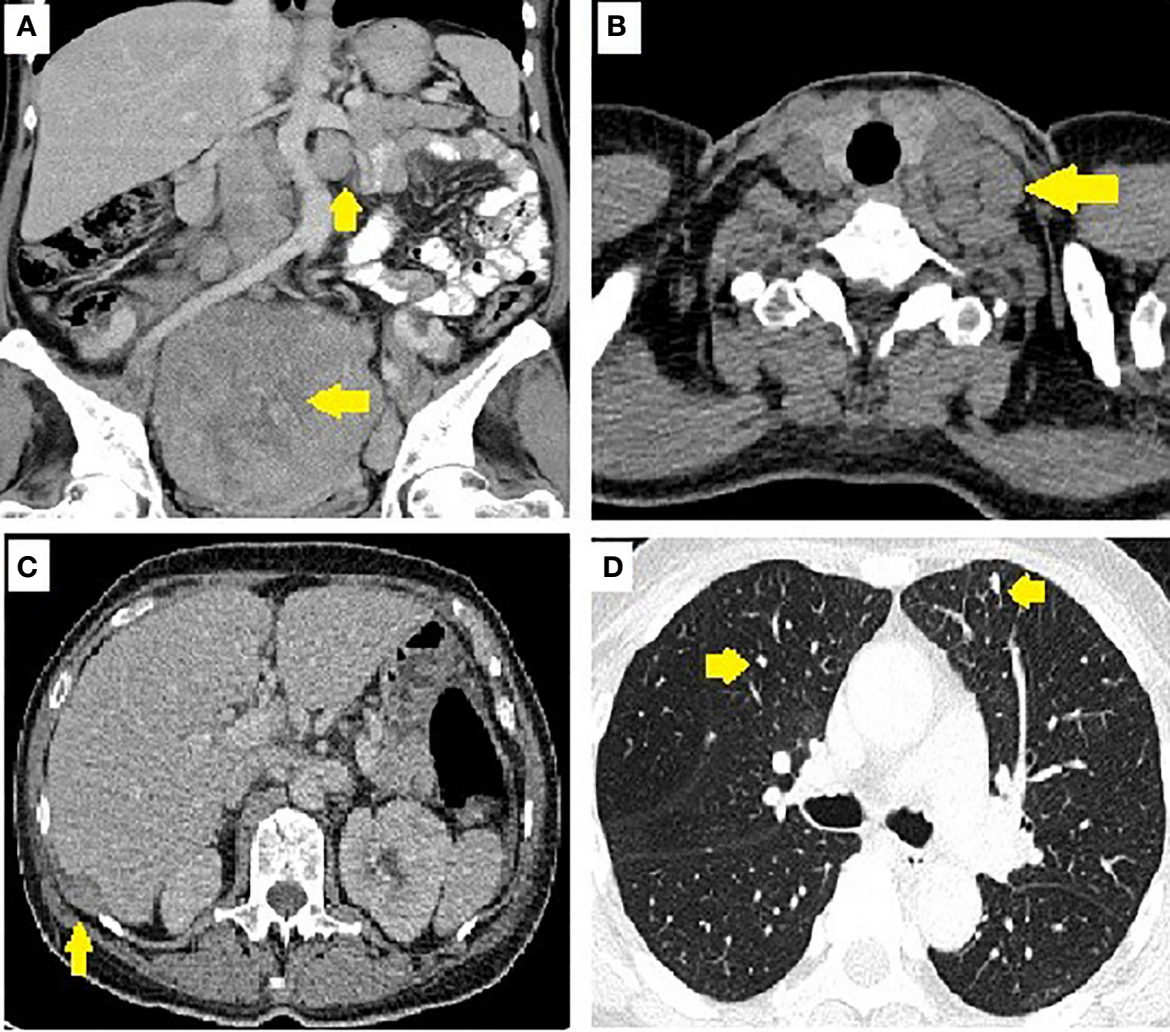
Image of pelvic tumor and metastasis. **(A)** Para-aortic lymph nodes and pelvic tumor. **(B)** Supraclavicular lymph nodes. **(C)** Sub-diaphragmatic seeding. **(D)** Lung metastasis.

Our patient was a 59-year-old woman who had no past medical history. She delivered three children and entered menopause at the age of 49 years. She complained of lower abdominal fullness for 6 months and increased abdominal circumference. She also experienced stress urinary incontinence, without dysuria, urinary frequency, or urinary urgency. She lost up to 3 kg of body weight in 1 month and had oedema in both feet. There was no fever, bowel habit change, nausea, vomiting, abdominal pain, or tarry/bloody stool. She visited a hospital where a giant pelvic mass was found. She was transferred to our hospital in April 2019. The initial laboratory work report is shown in **Table 1**. Profound elevated Lactate dehydrogenase (4123 IU/L, 98 – 192 IU/L), elevated Carbohydrate antigen 19-9 (39.34 U/mL, <37 U/mL) and Cancer antigen 125 (146.32 U/mL, <35 U/mL) was noted. Gynaecologic sonography showed a huge pelvic mass of approximately 22.5 × 13.3 × 16.1 cm in size. Computed tomography (CT) scans revealed a 23 × 17 × 21 cm heterogeneous mass occupying the lower abdomen and pelvic cavity with indistinct margin from the uterus and bilateral adnexa, suggestive of gynaecologic malignancy. There were multiple small peritoneal nodules, multiple enlarged para-aortic and bilateral iliac lymph nodes, and multiple small pulmonary metastases and lymph nodes over the left lower neck and left supra-clavicular regions (**Figure 1**). The visible liver and adrenal glands were unremarkable. She underwent optimal cytoreductive surgery for symptom relief. Left neck mass excision was performed. The initial pathological test report suggested suspected neuroendocrine carcinoma over bilateral ovaries, bilateral fallopian tubes, and the uterus, with omentum and lymph node metastasis. Right ovarian neuroendocrine carcinoma, stage IVB pT3cN1bM1, was the initially postulated diagnosis.

**Table 1:** Laboratory test for ovary tumor.

White blood cell count	8,500	(4,000–10,000)/µL
Hemoglobin	13.5	(11–16) g/dL
Platelet count	291,000	(140,000–450,000)/µL
Creatinine	0.8	(0.4–1.2) mg/dL
Alanine aminotransferase	31	(14–40) IU/L
Sodium	142	(136–144) mEq/L
Potassium	3.8	(3.5–5.1) mEq/L
Lactate dehydrogenase	4,123	(98–192) IU/L
Carcinoembryonic antigen	0.93	(<5.00) ng/mL
Alpha-Fetoprotein	2.66	(<10.00) ng/mL
Carbohydrate antigen 19-9	39.34	(<37.00) U/mL
Cancer antigen 125	146.32	(<35.00) U/mL

**Figure 2:**
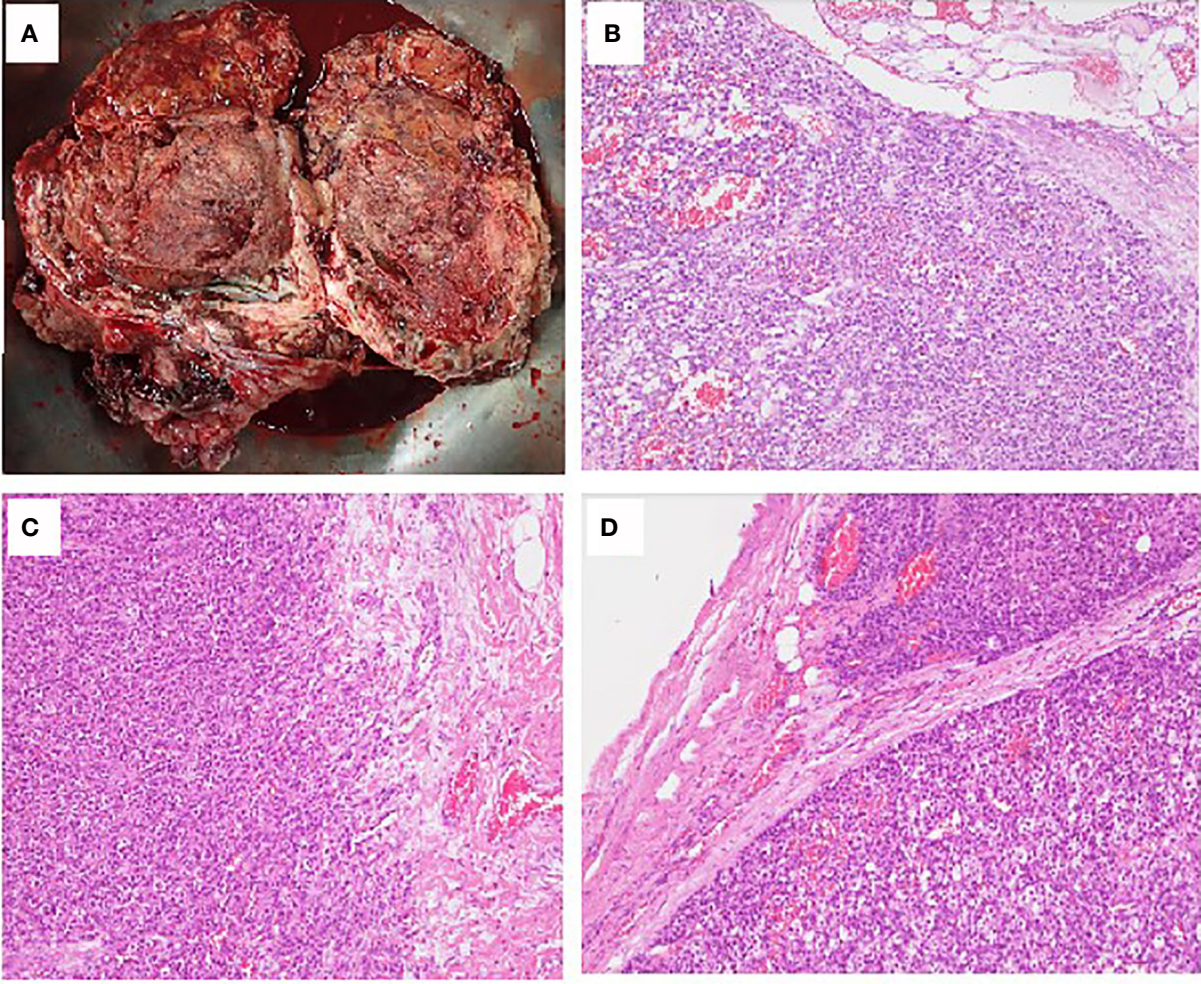
Pathological findings of ovarian adrenocortical carcinoma. **(A)** A piece of tissue measuring 23 × 17 × 10 cm in size. **(B)** Sections of the huge ovary and uterine body tumor showing solid sheets and nests of tumor cells with monotonous morphology with large, centrally located nuclei and abundant cytoplasm. Focal tumor necrosis is present. Lymphovascular invasion is prominent. **(C)** Biopsy sample of the peritoneum cavity. **(D)** Lymph node metastasis: Lesion cells are arranged in thick trabeculae and in organoid pattern. They contain eosinophilic cytoplasm and small dark nuclei. High prevalence of mitotic figures is seen.

**Figure 3:**
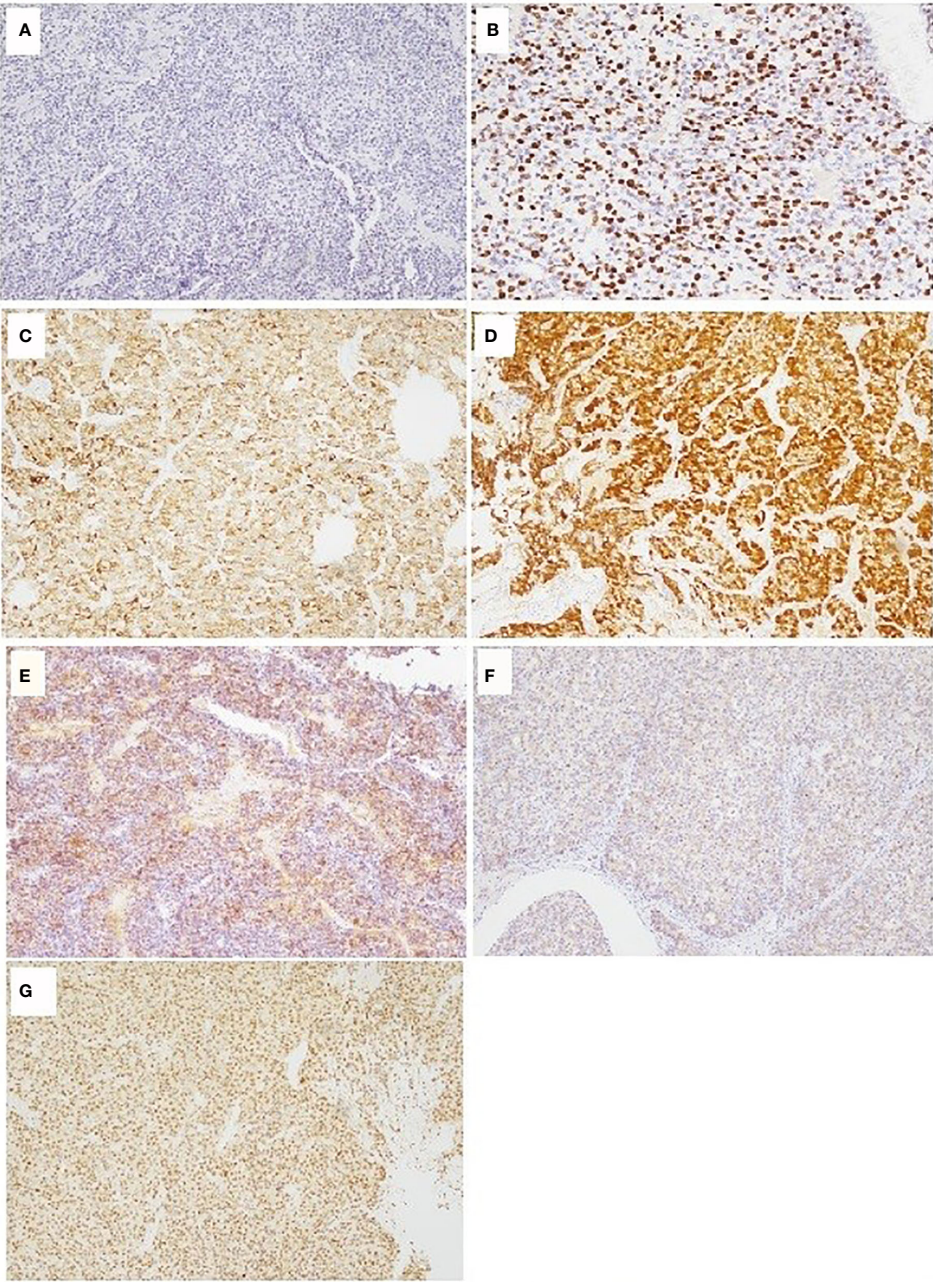
Immunohistochemistry of ovarian adrenocortical carcinoma. **(A)** Chromogranin A 100×: negative; **(B)** Ki-67 200×: high Ki-67-labeling proliferating index (>80%); **(C)** Alpha-inhibin 100×: positive; **(D)** Calretinin 100×: focally positive; **(E)** CD56 100×: focally positive; **(F)** Synaptophysin 100×: focally positive; **(G)** TFE-3 100×: positive.

She underwent postoperative chemotherapy with triweekly Etoposide (100mg/m^2^) and Cisplatin (100mg/m^2^). After consultation and discussion with another pathologist, the final pathological test report 2 months later showed ACC, probably arising from the adrenal cortical rest (**Figures 2** and **3**). Weiss score was 8 after discussion with pathologist. Hypertension with normal potassium level was noted during admission. She took amlodipine 5mg per day. The endocrine profile is listed in **Table 2**, which revealed normal aldosterone (12.8 ng/dL, 4.83–27 ng/dL) and decreased plasma renin activity (0.14 ng/mL/hr, 0.6–4.18 ng/mL/hr) level. ARR ratio was 91.4. Hence, ectopic primary aldosteronism could not be excluded. However, we did not perform saline infusion test or captopril test at that time because she was receiving chemotherapy and suffered from abdomen fullness, nausea and vomiting. She received dexamethasone as support therapy during chemotherapy. Leukopenia (900/µL) was noted. Cortisol and ACTH level was checked on the same day. Hence, the normal cortisol (10.54 μg/dL, 9.52–26.21 µg/dL) with elevated ACTH (70.26 pg/mL, 10–70 pg/mL) may be interpreted as acute illness. Follow up cortisol and ACTH level was 17.08 μg/dL and 20.15 pg/mL, respectively.

**Table 2:** Hormone profile for ovarian adrenocortical carcinoma.

Cortisol	10.54	(9.52–26.21) µg/dL
Adrenocorticotropic hormone	70.26	(10–70) pg/mL
Plasma renin activity	0.14	(0.6–4.18) ng/mL/hr
Aldosterone	12.8	(4.83–27) ng/dL
Oestrogen	<10	(menopause <10–28) pg/mL
Testosterone	<0.1	(<0.95) ng/mL
Dehydroepiandrosterone sulphate	12.9	(3.70–242.4) µg/dL

She received six courses of etoposide and cisplatin regimen and six courses of bevacizumab (900mg). However, LDH level elevated after completion of chemotherapy. Follow-up CT 6 months after the operation showed disease progression, with enlarged left supraclavicular and retrocaval lymph nodes, increased size of lung metastasis, and increased size and number of liver metastases and peritoneal seedings. She received mitotane 500mg per day 6 months after operation and mitotane was titrated to 1000mg per day. She used mitotane intermittently due to nausea, vomiting, poor appetite and dizziness. She was admitted to our hospital several times for poor appetite, vaginal bleeding, anaemia, and renal failure. She expired 9 months after cytoreductive surgery.

## Discussion

We presented the case of a 59-year-old woman who was diagnosed with ectopic ACC in the ovary. Initial pathological studies revealed the presence of local tumor necrosis and prominent lympho-vascular invasion. The Ki-67 proliferation labelling index was very high (>80%). Immunohistochemically, tumor cells were focally positive for CD56 and synaptophysin but also focally positive for calretinin. Two months later, the pathologist confirmed the diagnosis of ectopic ACC based on positivity for synaptophysin, alpha-inhibin, TFE-3, calretinin (focal), and CD56 (focal) and a high Ki-67-labeling proliferating index (>80%), which was much higher than usual ACC. Melan-A and steroidogenic factor-1 (SF1) were not available in our hospital. Adrenal gland was unlikely to be the origin because there was no adrenal lesion identified in the serial image studies. Ectopic primary aldosteronism could not be excluded. However, we did not perform saline infusion test or captopril test at that time because she was receiving chemotherapy and suffered from abdomen fullness, nausea and vomiting. Hence, we would not define whether this ectopic ACC was functional or non-functional, which was our limitation. Mitotane was immediately considered when ectopic ACC was diagnosed. However, project application was required in Taiwan before we started mitotane. Despite that adjuvant radiotherapy (RT) is important for local tumor control (9), RT was not considered due to multiple distant metastasis in our patient.

To our knowledge, there were only two reported cases of ectopic adrenocortical carcinoma in the ovary. Chentli presented the case of a 34-year-old female referred for Cushing’s syndrome in the postpartum period. The adrenal origin of the ectopic tissue was confirmed by immunostaining positivity to inhibin-α, melan-A, steroidogenic factor-1 (SF1), and synaptophysin (10). In another study, a 4-year-old girl initially presented with signs of rapid (within 1 month) early puberty (breast development plus pubic and armpit hair) (11). A right ovary mass was noted when she was 15 years old. Pathological examination revealed a 20-cm ovarian steroid tumor. At 21 years of age, pulmonary metastasis was detected. Complete remission of lung metastasis was achieved 5 years after mitotane initiation. The medical history was suggestive of a slowly progressive tumor. The patient was alive for 10 years after the initial operation (11).

Adrenal tumors in the ovary may originate from transformation of the adrenocortical embryonic remnants that break off during adrenal migration or descent of gonadal cells (12). Ectopic adrenal adenomas/carcinomas may result from the mutation of ovarian tissue and its steroid enzymes and/or acquisition of aberrant receptors (13). This pathological situation induces the ovary to synthesize adrenal hormones (14). Steroidogenic factor 1 (SF-1) is the most valid marker of ACC (15). Ki-67 can help define the diagnosis and prognosis of ACC. The general agreement is that ACCs have a Ki-67 labelling index of ≥5% (16). Ki-67 is a powerful prognostic marker in both localized and metastatic ACC (17–19). Positivity for steroid receptor coactivator-1 (SRC-1), inhibin, calretinin, synaptophysin, and melan A and negativity for Pax8, chromogranin, and high-molecular weight cytokeratin (HMWCK) on immunohistochemistry (IHC) studies may help distinguish ACC from other tumors (20).

The 5-year survival of patients with ACC is, respectively, 60–80%, 35–50%, and 0–28% in cases of tumors confined to the adrenal space, locally advanced disease, and metastatic disease (21). There is limited knowledge on the incidence and prognosis of ectopic ACC. In previous studies, the 5- and 10-year survival of patients who underwent resection of ectopic ACC was 26–38% and 7%, respectively (22–24). Surgical resection is the mainstay of treatment (22, 25). Patients with early mortality were found to have high rates of cortisol-secreting tumors and positive resection margins and high disease stages with nodal or synchronous distant metastasis (22–24). The importance of surgery was further confirmed by long-term survival attained with repeat resection of local or distant tumor recurrence (22). According to the presented cases, diagnosis of ectopic ACC is challenging; 50–60% of patients with ACC show clinical hormone excess (21). Hypercortisolism or mixed Cushing’s and virilizing symptoms are observed in the majority of these patients (21).

## Conclusion

We presented a rare case of ectopic ACC in the ovary. ACC is a rare disease, and ectopic ACC is even rarer. There is limited knowledge of its incidence and prognosis. When encountering hypercortisolism or mixed Cushing’s and virilizing symptoms without detectable adrenal nor pituitary tumors, ectopic tumor should be suspected. On the other hand, when pathological tests reveal atypical neuroendocrine feature of the organ, further IHC staining should be performed to rule out ectopic ACC. Whether the ectopic ACC is functional or not requires complete survey.

## Data Availability Statement

The original contributions presented in the study are included in the article/supplementary material. Further inquiries can be directed to the corresponding author.

## Ethics Statement

The studies involving human participants were reviewed and approved by Mackay Memorial Hospital. Written informed consent for participation was not required for this study in accordance with the national legislation and the institutional requirements. Written informed consent was not obtained from the individual(s) for the publication of any potentially identifiable images or data included in this article.

## Author Contributions

W-HT: writing and literature search. T-CC: medical and surgical practices. S-HD: analysis and interpretation. Y-HZ: concept, design, and medical practice. All authors contributed to the article and approved the submitted version.

## Conflict of Interest

The authors declare that the research was conducted in the absence of any commercial or financial relationships that could be construed as a potential conflict of interest.
